# Modulation of Gut Microbiota and Oxidative Status by β-Carotene in Late Pregnant Sows

**DOI:** 10.3389/fnut.2020.612875

**Published:** 2020-12-14

**Authors:** Xupeng Yuan, Jiahao Yan, Ruizhi Hu, Yanli Li, Ying Wang, Hui Chen, De-Xing Hou, Jianhua He, Shusong Wu

**Affiliations:** ^1^College of Animal Science and Technology, Hunan Agricultural University, Changsha, China; ^2^Hunan Xinguang'an Agricultural Husbandry Co., Ltd., Changsha, China; ^3^Department of Food Science and Biotechnology, Faculty of Agriculture, Kagoshima University, Kagoshima, Japan

**Keywords:** β-carotene, nitric oxide, antioxidant, gut microbiota, pregnant sows

## Abstract

Recent evidences suggest that gut microbiota plays an important role in regulating physiological and metabolic activities of pregnant sows, and β-carotene has a potentially positive effect on reproduction, but the impact of β-carotene on gut microbiota in pregnant sows remains unknown. This study aimed to explore the effect and mechanisms of β-carotene on the reproductive performance of sows from the aspect of gut microbiota. A total of 48 hybrid pregnant sows (Landrace × Yorkshire) with similar parity were randomly allocated into three groups (*n* = 16) and fed with a basal diet or a diet containing 30 or 90 mg/kg of β-carotene from day 90 of gestation until parturition. Dietary supplementation of 30 or 90 mg/kg β-carotene increased the number of live birth to 11.82 ± 1.54 and 12.29 ± 2.09, respectively, while the control group was 11.00 ± 1.41 (*P* = 0.201). Moreover, β-carotene increased significantly the serum nitric oxide (NO) level and glutathione peroxidase (GSH-Px) activity (*P* < 0.05). Characterization of fecal microbiota revealed that 90 mg/kg β-carotene increased the diversity of the gut flora (*P* < 0.05). In particular, β-carotene decreased the relative abundance of Firmicutes including *Lachnospiraceae* AC2044 group, *Lachnospiraceae* NK4B4 group and *Ruminococcaceae* UCG-008, but enriched Proteobacteria including *Bilophila* and *Sutterella*, and Actinobacteria including *Corynebacterium* and *Corynebacterium* 1 which are related to NO synthesis. These data demonstrated that dietary supplementation of β-carotene may increase antioxidant enzyme activity and NO, an important vasodilator to promote the neonatal blood circulation, through regulating gut microbiota in sows.

## Introduction

Beta-(β-)carotene is a widely distributed phytochromes (1), and is generally considered as a precursor of vitamin A (2, 3). It belongs to the fat-soluble substance which is incorporated into chylomicrons and absorbed in the intestine through passive diffusion (4). When ingested into the intestine, β-carotene is partially transformed into vitamin A, and the remaining β-carotene is passed by blood circulation to target organs such as the liver, ovary, adipose tissue and adrenal gland (5). Around 17–45% of β-carotene can be absorbed into the circulatory system (1). The importance of vitamin A in female reproductive health has been well-documented (6). Previous studies have found a high concentration of β-carotene in the luteum, and the lack of β-carotene results in delayed ovulation, luteal phase defect, and increased risk of ovarian cyst (1, 7). Moreover, the secretion of pregnancy hormones is linked to serum β-carotene concentrations, indicating that β-carotene can play an important role in reproduction (8, 9).

Multiple studies have shown that low-level inflammation, progressive oxidative stress, and metabolic disorders occur during the perinatal period. During pregnancy, the rapid cell proliferation of the uterus and the placenta, growth of the fetus, and childbirth will progressively increase the reactive oxygen species (ROS) and decrease the antioxidant capacity of the body (10). The pro-inflammatory interleukins (ILs) such as IL-6 can be substantially increased in maternal serum as pregnancy progresses (11). Furthermore, insulin resistance during pregnancy will decrease glucose utilization, while excessive energy intake and obesity during pregnancy will exacerbate maternal inflammation and oxidants stress, thereby triggering insulin resistance and having adverse effects on pregnancy (12). Recent studies have shown that significant change in the gut microbiota during different periods of pregnancy can affect the physiological state and metabolic process of host, indicating that gut microbiota plays a vital role during pregnancy (13, 14). In modern pig farming industry, constipation occurs frequently in pregnant sows due to intestinal disorder, which is largely determined by the dysfunction of gut microbiota. β-carotene has been considered as a potent inhibitor of oxidative stress and inflammation both *in vitro* and *in vivo* (15, 16), and it can suppress the expression of proinflammatory cytokines such as IL-1β and IL-6 to alleviate inflammation and oxidative stress induced by ischemia injury (17). However, it is not clear whether β-carotene exerts biological functions by modulating the gut microbiota.

Therefore, this study aimed to investigate the effect of β-carotene on the reproductive performance of sows, and challenged to clarify the potential mechanisms from the aspect of gut microbiota.

## Materials and Methods

### Experimental Design and Diets

The animal model and experimental procedures used in this experiment were approved by the Hunan Agricultural University Institutional Animal Care and Use Committee (No. 201903). A total of 48 hybrid pregnant sows (Landrace × Yorkshire), with similar parity (3–7 fetuses) were used in this study. The experimental animals which kept in gestation stalls with fully slatted floors measuring under environment temperature and had free access to water, were randomly allocated into three treatments (*n* = 16). Based on previous studies, sows in the treatments were fed with a basal diet (control group, CTL), a diet containing 30 mg/kg of β-carotene (β-carotene low dose group, CAR-L), or a diet containing 90 mg/kg of β-carotene (β-carotene high dose group, CAR-H), respectively, based on previous studies (8, 18). The sows were fed at 6:00 a.m., 12:00 p.m., and 6:00 p.m. with ~3.2 kg of feed/sow/day. The experiment started on day 90 of gestation and continued until delivery. The composition of the basal diet, which meets the nutritional requirements of pigs according to NRC (2012), was shown in Supplementary Table 1.

### Reproductive Performance Markers

The number of live birth, litter weight at parturition and average weight of piglets born alive were measured within 24 h after farrowing.

### Sample Collections

At the day of parturition, blood samples (5 mL) were collected from the marginal auricular vein into anticoagulant-free vacuum tubes and centrifuged on 1,500 × g for 10 min after standing at room temperature for 30 min to get the serum. Fecal samples (around 2 g from each sow) were collected from the innermost of feces into sterile tubes after defecation in the morning, and snap-frozen in liquid nitrogen before storage at −80°C for further DNA extraction.

### Measurement of Serum Biochemical Indices

Total antioxidant capacity (T-AOC), total superoxide dismutase (T-SOD) activity, glutathione peroxidase (GSH-Px) activity, the level of thiobarbituric acid reactive substances (TBARS), and the nitric oxide (NO) level were determined in serum by using respective assay kits (Nanjing Jiancheng Bioengineering Institute, Nanjing, China) according to the manufacturer's instructions as described previously (19). The levels of glucose (GLU, 0.06–27.8 mmol/L), total protein (TP, 1.74–100 g/L), total cholesterol (TC, 0.09–25.85 mmol/L), triglycerides (TG, 0.05–11.3 mmol/L), high-density lipoproteins (HDL-c, 0.065–3.8 mmol/L), low-density lipoprotein cholesterol (LDL-c, 0.2–12 mmol/L), immunoglobulin G (IgG, 0.25–35 g/L) and immunoglobulin M (IgM, 0.25–5.00 g/L) were measured with respective kits from Mindray Medical International Ltd., China by using an automated biochemical analyser BS-200 (Mindray, China).

### Characterization of Gut Microbiota

Fecal microbiota was characterized by 16S rRNA gene sequencing. Briefly, total DNA was extracted from fecal samples (six random samples from each group) by using a DNA Isolation Kit (MoBio Laboratories, Carlsbad, CA, USA) following the manufacturer's manual. Purity and quality of the genomic DNA were checked on 0.8% agarose gels. The V3–4 hypervariable region of the bacterial 16S rRNA gene was amplified with the primers 338F (5′-ACTCCTACGGGAGGCAGCA-3′) and 806R (5′-GGACTACHVGGGTWTCTAAT-3′). For each sample, 10-digit barcode sequence was added to the 5′ end of the forward and reverse primers (provided by Allwegene Technology Inc., Beijing, China). The PCR was carried out on a Mastercycler Gradient (Eppendorf, Germany) using 25 μL reaction volumes, containing 12.5 μL KAPA 2G Robust Hot Start Ready Mix, 1 μL Forward Primer (5 μmol/L), 1 μL Reverse Primer (5 μmol/L), 5 μL DNA (total template quantity is 30 ng), and 5.5 μL H_2_O. Cycling parameters were 95°C for 5 min, followed by 28 cycles of 95°C for 45 s, 55°C for 50 s, and 72°C for 45 s with a final extension at 72°C for 10 min. Three PCR products per sample were pooled to mitigate reaction-level PCR biases. The PCR products were purified using a QIAquick Gel Extraction Kit (QIAGEN, Germany), and quantified using Real Time PCR, and sequenced on Miseq platform at Allwegene Technology Inc., Beijing, China. After the run, image analysis, base calling and error estimation were performed using Illumina Analysis Pipeline Version 2.6. The raw data were first screened and sequences were removed from consideration if they were shorter than 200 bp, had a low quality score (≤ 20), contained ambiguous bases or did not exactly match to primer sequences and barcode tags. Qualified reads were separated using the sample-specific barcode sequences and trimmed with Illumina Analysis Pipeline Version 2.6. And then the dataset was analyzed using QIIME (Version 1.8.0). The sequences were clustered into operational taxonomic units (OTUs) at a similarity level of 97%, to generate rarefaction curves and to calculate the richness and diversity index. The Ribosomal Database Project (RDP) Classifier tool was used to classify all sequences into different taxonomic groups.

### Statistical Analysis

Results were expressed as means ± SD. The significant differences between groups were analyzed by one-way analysis of variance tests, followed by Fisher's least significant difference (LSD) and Duncan's multiple range tests with the SPSS statistical program (SPSS19, IBM Corp., Armonk, NY, USA). A probability of *P* < 0.05 was considered significant.

## Results

### The Influence of β-Carotene on the Reproductive Performance of Sows

As shown in Table 1, the number of live birth was increased to 11.82 ± 1.54 and 12.29 ± 2.09 in CAR-L (30 mg/kg β-carotene) group and CAR-H (90 mg/kg β-carotene) group respectively, while the CTL group was 11.00 ± 1.41, although there had no statistical significance (*P* = 0.201). The litter weight at parturition showed a similar trend with the number of live birth, and the average weight of piglets born alive kept no change.

**Table 1:** The effect of β-carotene on the reproductive performance of sows.

**Item**	**CTL**	**CAR-L**	**CAR-H**	***P-*value**
Total born number	11.82 ± 1.72	12.55 ± 1.37	13.17 ± 2.29	0.233
The number of live birth	11.00 ± 1.41	11.82 ± 1.54	12.29 ± 2.09	0.201
Litter weight at parturition, kg	16.16 ± 3.72	17.26 ± 4.07	17.13 ± 2.97	0.729
Average weight of piglets born alive, kg	1.42 ± 0.19	1.46 ± 0.27	1.41 ± 0.21	0.844
Stillborn piglets number	9	8	9	-

*CTL, a basal diet; CAR-L, a basal diet containing 30 mg/kg β-carotene; CAR-H, a basal diet containing 90 mg/kg β-carotene. Data were shown as means ± SD (n = 16)*.

### The Effect of β-Carotene on Serum Biochemical Markers in Sows

**Figure 1:**
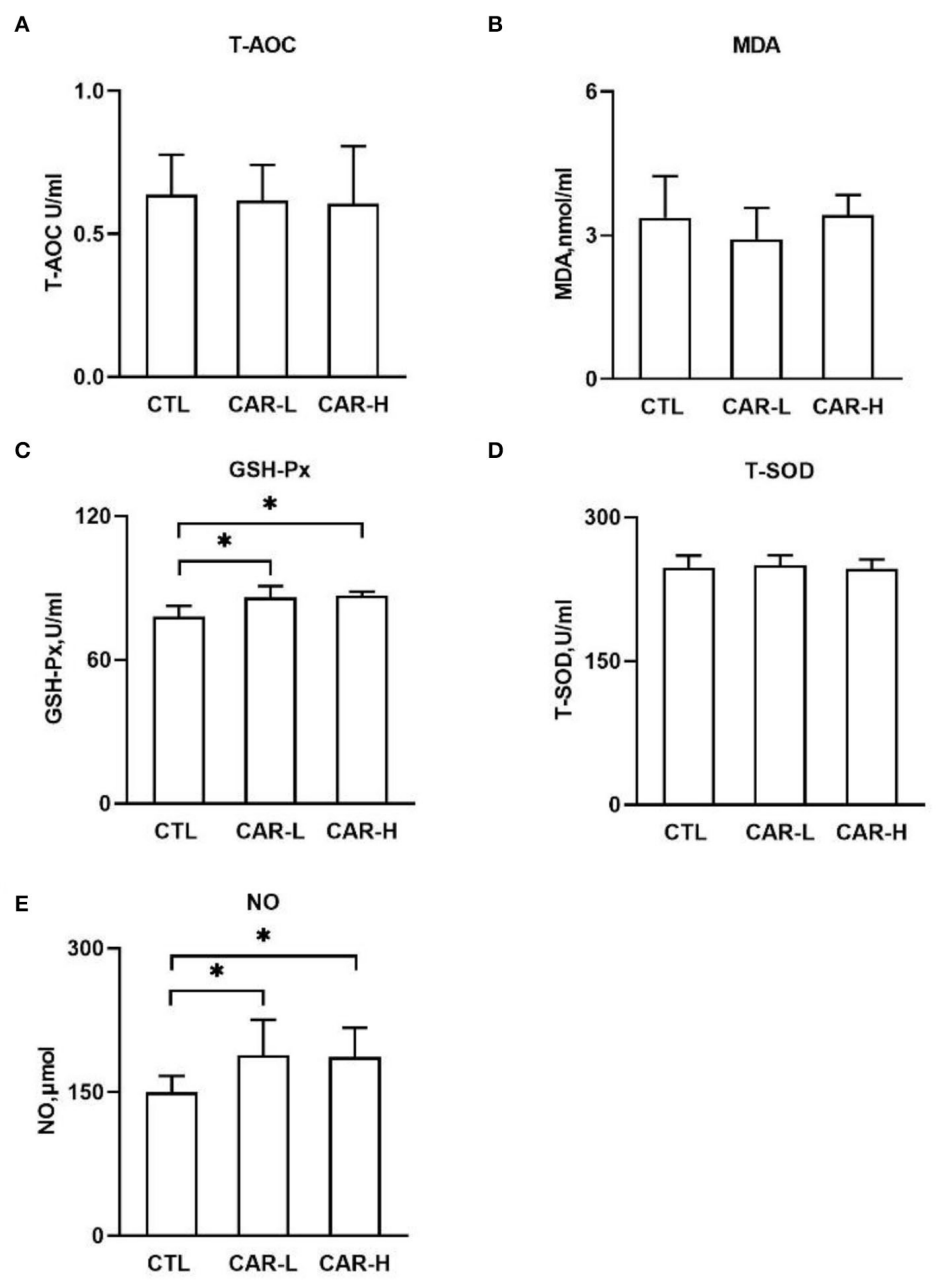
The effect of β-carotene on serum levels of antioxidant indicators and NO. Serum levels of T-AOC **(A)**, TBARS **(B)**, GSH-Px **(C)**, T-SOD **(D)**, and NO **(E)** were determined by using respective kits. CTL, a basal diet; CAR-L, a basal diet containing 30 mg/kg β-carotene; CAR-H, a basal diet containing 90 mg/kg β-carotene. Data were shown as means ± SD (*n* = 16), *P < 0.05.

As progressive oxidative stress plays a negative role during the perinatal period, the oxidative stress markers including T-AOC, TBARS, GSH-Px, and T-SOD were measured in the serum of sows. As shown in Figure 1, the activity of GSH-Px (C) was increased significantly (*P* < 0.05) in both CAR-L (85.99 ± 4.75 U/mL) and CAR-H (86.8 ± 1.67 U/mL) group, as compared with CTL group (77.94 ± 4.54 U/mL). However, no significant differences were observed in the levels of T-AOC (A), MDA (B), and T-SOD (D) among the three groups (*P* > 0.05).

Nitric oxide (NO) is a signaling molecule involved in oxidative stress and inflammation, and also considered as an important vasodilator to promote the neonatal blood circulation (14). Thus, NO was further measured in serum, and the results showed that the level of NO was increased to 188.33 ± 37.13 μmol and 186.95 ± 30.06 μmol in CAR-L and CAR-H group respectively, which were significantly (*P* < 0.05) higher than that in CTL group (149.54 ± 17.12 μmol) (Figure 1E).

In addition, indicators of immune response and glucolipid metabolism were also measured in serum due to their important roles in pregnancy. However, β-carotene had no significant effects on serum levels of immunoglobulins (IgM & IgG), lipids (Tc, Tg, HDL-c & LDL-c), Glu, and Tp (Supplementary Table 2).

### Modulation of Gut Microbiota by β-Carotene

**Figure 2:**
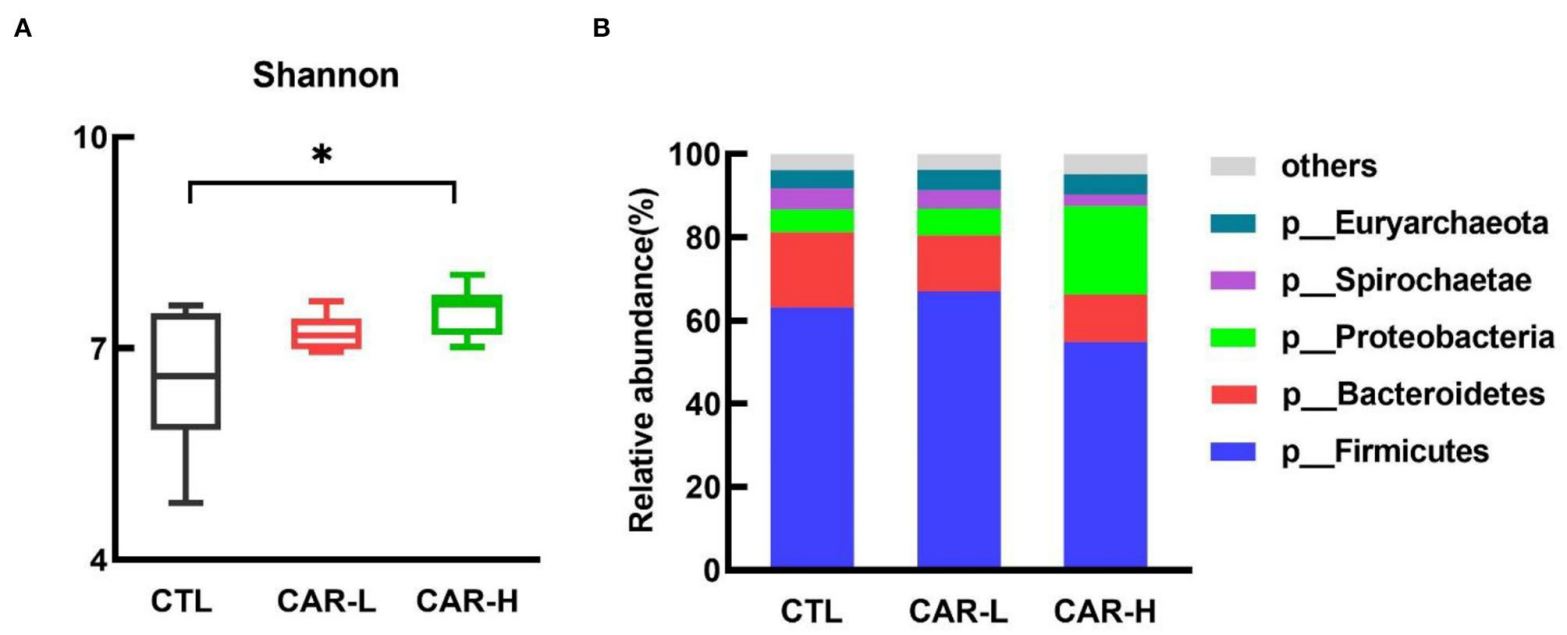
The effect of β-carotene on fecal microbial community. The effect of β-carotene on the Shannon index **(A)** and the relative abundances of microbial phyla **(B)** were analyzed by 16S rRNA gene sequencing. CTL, a basal diet; CAR-L, a basal diet containing 30 mg/kg β-carotene; CAR-H, a basal diet containing 90 mg/kg β-carotene. Data were shown as means ± SD (*n* = 6), *P < 0.05.

To gain an insight into the effect of β-carotene on gut microbiota, the composition and relative abundance of fecal microbiota was characterized by using high throughput 16S rRNA gene sequencing. As shown in Figure 2, supplementation of β-carotene dose-dependently increased the Shannon index (A) of the microbiota, and a significant difference was observed between CTL and CAR-H group (*P* < 0.05). Further analysis on the relative abundance of bacterial phyla showed that the microbial community was dominated by Firmicutes, Bacteroidetes, Proteobacteria, Spirochaetae, and Euryarchaeota, which account for 97% of total microbes, and supplementation of β-carotene increased the relative abundance of Proteobacteria.

**Figure 3:**
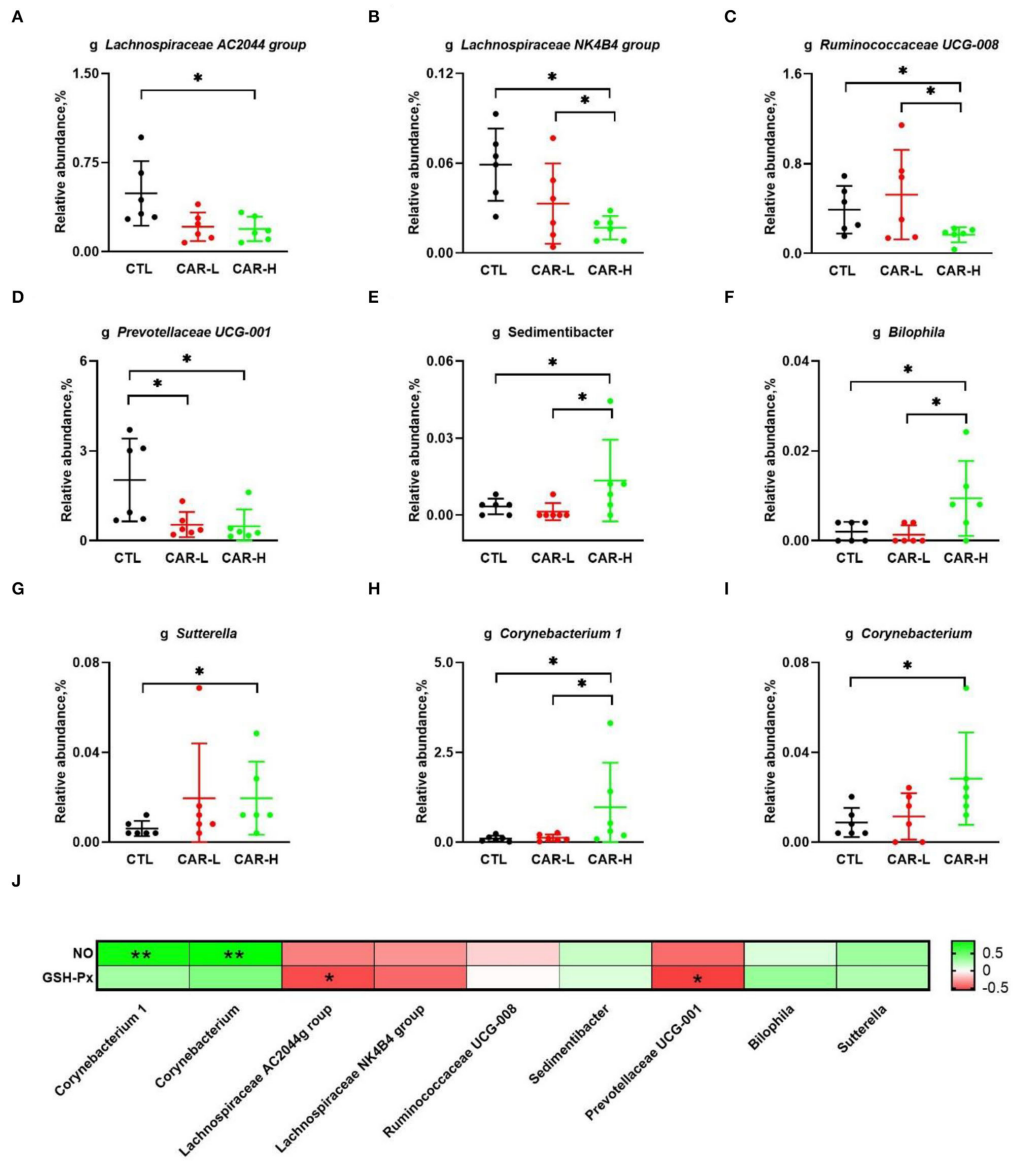
Modulation of fecal microbiota by β-carotene at the genus level. The relative abundance of microbial genera including *Lachnospiraceae* AC2044 group **(A)**, *Lachnospiraceae* NK4B4 group **(B)**, *Ruminococcaceae* UCG-008 **(C)**, *Prevotellaceae UGC-001* **(D)**, *Sedimentibacter* **(E)**, *Bilophila* **(F)**, *Sutterella* **(G)**, *Corynebacterium* 1 **(H)**, and *Corynebacterium* **(I)** in each group were characterized by 16S rRNA gene sequencing, and the correlation between NO, GSH-Px and the changed gut microbiota **(J)** were analyzed by Spearman's correlation analysis. The intensity of the colors represent the degree of association (red, negative correlation; green, positive correlation). Significant correlations were marked by *P < 0.05, **P < 0.01. CTL, a basal diet; CAR-L, a basal diet containing 30 mg/kg β-carotene; CAR-H, a basal diet containing 90 mg/kg β-carotene. Data were shown as means ± SD (*n* = 6), *P < 0.05.

At the genus level, a total number of 299 microbial genera were analyzed, and nine genera were found to be significantly different among the three groups. As shown in Figure 3, β-carotene decreased the relative abundances of *Lachnospiraceae AC2044 group* (A), *Lachnospiraceae NK4B4 group* (B), and *Ruminococcaceae UCG-008* (C), which belong to the phylum of Firmicutes. Meanwhile, β-carotene reduced the relative abundance of *Prevotellaceae UCG-001* (D), a genera belong to the phylum of Bacteroidete. On the other hand, β-carotene increased the relative abundance of *Sedimentibacter* (E) belonging to the phylum of Firmicutes, *Bilophila* (F) and *Sutterella* (G) belonging to the phylum of Proteobacteria, as well as *Corynebacterium 1* (H) and *Corynebacterium* (I) belonging to the phylum of Actinobacteria. Based on the results of gut microbiota and serum biochemical markers, the correlation between GSH-Px, NO and the changed microbial genera were further analyzed. The results showed that *Corynebacterium 1* and *Corynebacterium* were positively correlated (*P* < 0.01) with the NO level, while *Prevotellaceae UCG-001 and Lachnospiraceae AC2044 group* were negatively correlated (*P* < 0.01) with the GSH-Px level (J).

## Discussion

It has been reported that injection or dietary supplementation of β-carotene during early gestation can enhance embryo survival, litter size and litter weight of piglets (9, 20, 21), but less information on its impact in late pregnancy has been obtained. However, in this study, dietary supplementation of β-carotene at late stage of pregnancy showed limited effect on the number of live birth and litter weight at parturition. During late pregnancy, maternal metabolism will be increased to adapt the nutritional requirements of fetal growth and placenta metabolism, which usually manifested by the increase in TG, TC, HDL-c and LDL-c levels (22–24). However, supplementation of β-carotene had no significant effect on the serum levels of lipids (TC, TG, HDL-c, and LDL-c) and immunoglobulins (IgM & IgG) in sows, which suggesting that β-carotene has limited effects on lipid metabolism and immunoglobulin production. Oxidative stress has a negative effect on oocyte maturation, ovulation, implantation and blastocyst formation (25). In the perinatal period of mammals, fetal growth, lactation and increased metabolism can induce the production of progressive ROS, and negatively impact sow reproductive performance including reduces litter size, survival ratio of piglets and the ability for lactation (10, 22). Our results revealed that β-carotene significantly increased the activity of GSH-Px, an antioxidant enzyme against ROS. The findings were consistent with a previous cell model analysis in which β-carotene increased Nrf2 expression (2). Moreover, adequate uterine blood flow throughout gestation is reported to be essential for placental and fetal growth, especially in the late phase of pregnancy (26), as the increased blood flow velocity in placenta can enhance fetus to uptake nutrients absorption (10, 26). As a signaling molecule that conveys information between cells, NO is considered to be an important vasodilator to promote the neonatal blood circulation (27). Thus, serum NO levels in the sow serum were further tested, and the findings showed that β-carotene substantially increased NO levels, implying that β-carotene has a possible beneficial effect on uterine and placenta blood circulation.

Gut microbiota plays an essential role in the regulation of nutrient utilization and metabolism (28), and the microbial diversity can be used as a biomarker to reflect health and metabolic capacity of animals (14). A previous study has also pointed out that gut microbiota is closely related to the reproductive performance of sows (13). The intestinal microbial composition changes significantly during pregnancy, and α-diversity will gradually increase throughout the lactation period (29). Furthermore, the structure of intestinal flora differ at different stages of pregnancy, and enrichment of α-diversity can enhance metabolic capacity and increase the flux of nutrients to the fetus for growth and development (30). Clarke et al. (14) revealed that people with rapid metabolism show a higher α-diversity of gut microbiota. In this study, β-carotene increased the Shannon index, which suggested that it may enhance the metabolic capacity and promote fetal development. Further analysis at the genus level revealed that β-carotene mainly down-regulated the relative abundance of genera *Lachnospiraceae AC2044 group, Lachnospiraceae NK4B4 group*, and *Ruminococcaceae UCG-008* belonging to the phylum of Firmicutes, as well as *Prevotellaceae UCG-001* belonging to the phylum of Bacteroidete. On the other hand, β-carotene enriched *Sutterella* and *Bilophila* belonging to the phylum of Proteobacteria, *Corynebacterium 1* and *Corynebacterium* belonging to the phylum of Actinobacteria, as well as *Sedimentibacter* belonging to the phylum of Firmicutes. *Lachnospiraceae* and *Ruminococcaceae* are closely related to the production of butyrate (31, 32). *Prevotellaceae UCG-001* belongs to the family of *Prevotellace* showed a positive correlation with the expression of inflammatory factors in our recent study (33). *Sutterella* is positively correlated with neutral detergent fiber (NDF) digestibility (34), and *Bilophila* has a positive correlation with obesity-related markers (18), while *Sedimentibacter* can secrete cellulase enzyme to digest cellulose into glucose (19, 34). Our results revealed that *Corynebacterium 1 and Corynebacterium* were positively correlated with NO level, while *Prevotellaceae UCG-001 and Lachnospiraceae AC2044 group* were negatively correlated with GSH-Px level. Other studies have also shown that *Corynebacterium* can induce the expression of NO synthase in a number of tissues in mice, and the decrease in blood pressure induced by *Corynebacterium* is associated with the induction of NO synthase (35). This can partially explain the increased NO level induced by β-carotene in this study. A recent study has also indicated that the abundance of *Corynebacterium* is increased with reproductive performance (36). Therefore, β-carotene potentially enhanced NO production by up-regulating the relative abundance of *Corynebacterium*, although more direct evidences are need.

## Conclusions

In conclusion, dietary supplementation of β-carotene showed limited effect on the reproductive performance of sows but increased the activity of GSH-Px and NO production. Analysis on fecal microbiota revealed that β-carotene may increase the diversity of the microbial flora with enriched *Bilophila, Sutterella, Sedimentibacter, Corynebacterium 1* and *Corynebacterium* which related to the synthesis of NO, an important vasodilator that can promote the neonatal blood circulation.

## Data Availability Statement

The raw data supporting the conclusions of this article will be made available by the authors, without undue reservation.

## Ethics Statement

The animal study was reviewed and approved by Hunan Agricultural University Institutional Animal Care and Use Committee.

## Author Contributions

XY and JY were the primary investigator in this study. RH, YL, and YW participated in the animal experiments. HC participated in sample analysis. D-XH revised the manuscript. SW and JH designed this study and wrote the manuscript as corresponding author. The authors read and approved the final manuscript.

## Conflict of Interest

XY was employed by Hunan Xinguang'an Agricultural Husbandry Co., Ltd., and the sows for experiment were provided by this company. The remaining authors declare that the research was conducted in the absence of any commercial or financial relationships that could be construed as a potential conflict of interest.
