# Supplementary material for: Modulation of Gut Microbiota and Oxidative Status by β-Carotene in Late Pregnant Sows
Source: Front Nutr. 2020 Dec 14;7:612875. doi: 10.3389/fnut.2020.612875 (PMC7768031; doi:10.3389/fnut.2020.612875)
Supplement: Supplementary file 1 [file Data_Sheet_1.docx]

# Supplementary Material

Supplemental Table 1. Composition of the basal diet

| Ingredients | % |
| --- | --- |
| Corn | 56.9 |
| Rice bran | 8 |
| Soybean meal (CP 46%) | 12.5 |
| Soy oil | 1 |
| Soybean hull (CP 10%) | 18 |
| NaCl | 0.45 |
| Choline chloride (50%) | 0.16 |
| Limestone | 1.2 |
| CaHPO_4_ | 1.15 |
| NaHCO₃ | 0.3 |
| Lys，% | 0.1 |
| Thr，% | 0 |
| Vitamin Premix | 0.04 |
| Mineral Premix | 0.1 |
| Mildew Preventive | 0.1 |
| Total | 100 |
| DE，kcal/kg | 3070 |
| CP，% | 12.7 |
| Ca，% | 0.87 |
| Total P，% | 0.6 |
| Available P，% | 0.4 |
| L-Lys·HCL(98%)，% | 0.72 |
| Met，% | 0.22 |
| L-Thr(98%)，% | 0.52 |

The vitamin premix provided for per kg of feed: VA, 9920 IU; VD3, 1985 IU; VE, 66 IU; VK 4.4 mg; VB3, 44 mg; VB2, 10 mg; VB5, 33 mg; VB12, 37 mg; VB7, 220 mg; VB9, 1325 mg; VB1, 2.2 mg; VB6, 3.3 mg. The mineral premix provided for per kg of feed: Cu, 8 mg; Fe, 80 mg; Mn, 45 mg; Zn, 80 mg; I, 0.5 mg; Se, 0.3 mg.

**Supplemental Table 2.** The influence of β-carotene on serum markers of immunity and lipid metabolism

| Item | CTL | CAR-L | CAR-H | *P* value |
| --- | --- | --- | --- | --- |
| Glu(mmol/L) | 5.16±0.83 | 4.77±0.93 | 4.58±0.86 | 0.265 |
| Tp(g/L) | 65.09±4.06 | 61.75±3.41 | 65.73±3.95 | 0.085 |
| Tc(mmol/L) | 3.7±0.72 | 3.53±0.98 | 3.65±0.55 | 0.795 |
| Tg(mmol/L) | 0.48±0.18 | 0.44±0.11 | 0.49±0.17 | 0.795 |
| HDL-c(mmol/L) | 0.35±0.06 | 0.33±0.09 | 0.31±0.05 | 0.279 |
| LDL-c(mmol/L) | 0.5±0.14 | 0.43±0.12 | 0.45±0.08 | 0.280 |
| IgM(mg/L) | 0.69±0.12 | 0.66±0.12 | 0.65±0.16 | 0.862 |
| IgG(mg/L) | 0.74±0.23 | 0.89±0.13 | 0.81±0.25 | 0.288 |

CTL, a basal diet; CAR-L, a basal diet containing 30 mg/kg β-carotene; CAR-H, a basal diet containing 90 mg/kg β-carotene.
